# Understanding Suicidal Behavior: The Contribution of Recent Resting-State fMRI Techniques

**DOI:** 10.3389/fpsyt.2016.00069

**Published:** 2016-04-19

**Authors:** Gianluca Serafini, Matteo Pardini, Maurizio Pompili, Paolo Girardi, Mario Amore

**Affiliations:** ^1^Section of Psychiatry, Department of Neuroscience, Rehabilitation, Ophthalmology, Genetics and Maternal and Child Health, University of Genoa, Genoa, Italy; ^2^Section of Neurology, Department of Neuroscience, Rehabilitation, Ophthalmology, Genetics and Maternal and Child Health, University of Genoa, Genoa, Italy; ^3^Department of Neurosciences, Suicide Prevention Center, Sant’Andrea Hospital, University of Rome, Rome, Italy

**Keywords:** suicidal behavior, resting state, rsfMRI, functional deficits, brain networks

## Introduction: Suicidal Behavior and Resting-State fMRI Techniques

Suicidal behavior is a relevant and multifaceted public health issue and is commonly associated with a significant disability and psychosocial impairment. To date, no available biomarkers are able to predict which subjects will develop suicide over time, and this is hardly surprising given the number of factors that have been hypothesized to modulate suicide risk based on the current literature (1).

In the effort to solve this shortcoming, a possible approach is represented by the search of those patterns of brain activation that are associated with suicidal behavior and may be identified using functional neuroimaging techniques. To date, the most commonly used functional neuroimaging technique is represented by functional magnetic resonance imaging (fMRI). fMRI may detect the local changes in the relative concentrations of oxy- and deoxy-hemoglobin, induced by local metabolic demand [i.e., it measures the so-called blood–oxygen level-dependent (BOLD) signals] (2). fMRI data can be also acquired while the imaged subject is performing a given task (i.e., task-dependent fMRI) or at rest (resting-state fMRI – rsfMRI).

There are studies showing aberrant neural activity patterns in suicide attempters that were carried out using task-based BOLD fMRI (3). Indeed, task-based fMRI has been used to probe the neural substrates of specific cognitive and emotional intermediate phenotype of suicide, such as error monitoring (4) and decision-making (5), but task-based fMRI is inherently limited by the need of active collaboration by the scanned subject as well as by the nature of the task during fMRI data acquisition. fMRI data can be also acquired while the subject is not performing any task – i.e., at rest (rsfMRI) – to evaluate which brain regions present same patterns of activation over time that are supposed to represent a valid surrogate marker of functional connectivity between different gray matter areas and over the whole brain (6).

Compared with task-based fMRI, rsfMRI is not dependent on subject collaboration (except for the requirement to lay in the scanner as much as possible), thus increasing its inter-subject and intra-subject reproducibility. Moreover, rsfMRI allows to explore the resting-state brain networks, in particular, the default mode network (DMN), that have been reported to be altered in several psychopathological conditions and may be not easily investigated using the commonly available task-based fMRI (7, 8). Finally, as rsfMRI data can be analyzed over the whole brain, they do not require to have an *a priori* hypothesis regarding the involvement of specific brain regions.

## Can rsfMRI Inform about Suicidal Behavior?

In order to perform a critical overview of the existing studies about the main topic, a reference search was carried out across the Medline and ScienceDirect databases (January 1980 and February 2016). The search used the following terms: “Resting-state fMRI” OR “Resting-state functional magnetic resonance imaging” OR “rsfMRI techniques” AND “Suicid*” (including suicidal behavior OR suicide ideation OR suicidal thoughts OR deliberate self-harm OR suicidal attempt). In addition, the reference lists of all papers identified were reviewed, and imaging evidence investigating suicidality as a secondary emergence of disturbed experience of self in personality disorders have been also included.

Although fMRI may investigate brain activity both in resting conditions and during activation, in the present paper, we mainly focused on brain imaging in resting conditions estimating regional brain activity when environmental activation is standardized. fMRI studies that investigated depressed patients pointed to the possible role of a host of different regions in this complex construct. Interestingly, all these brain areas have been also shown to play a role in different psychopathological domains such as modulation of physiological responses to emotions, emotional dysregulation, and self-processing, which in turn are also supposed to play a role in the emergence of suicide behavior (3, 5). As reviewed by Desmyter and colleagues (9), the reduced perfusion of the prefrontal cortex in suicidal patients is a commonly observed finding in functional neuroimaging resting conditions.

Indeed, the search for the neural bases underlying the cognitive and emotional intermediate phenotypes may reveal interesting neurocognitive constructs concerning suicidality. Cao et al. (10), for example, aimed to explore changes in neural circuit organization associated with suicidal behavior and proposed that disruptions in fronto-limbic or fronto-parietal–cerebellar pathways may lead to poor executive functioning, lack of impulse control, cognitive inflexibility, and impaired decision-making in suicidal young adults.

Very recently, Northoff (11) reported that depressive symptoms may be interpreted as spatiotemporal disturbances of the resting-state activity and its spatiotemporal structure according to the general assumption that brain resting-state activity may be defined in a functional/physiological manner rather than anatomically/structurally. Based on this suggestion, ruminations and enhanced self-focus in depressed patients are referred to abnormal spatial organization of resting-state activity, whereas anhedonia and suicidal ideation may be associated with increased focus on the past and enhanced past-focus as basic temporal disturbances of the resting state. Aiming to investigate the neural correlates of suicide thoughts, fMRI during presentation of autobiographical memories in depressed patients who recently attempted suicide has been also carried out. A deactivation of frontal cortical areas has been observed in suicidal episodes (mental pain plus suicide action), whereas an increased neural activity in the medial prefrontal, the anterior cingulate cortex, and the hippocampus occurred during the recall of the suicide action compared to mental pain (12). The authors suggested that suicidal mode is a state-dependent phenomenon that can be triggered by a specific stimulus and it may possess the quality of a traumatic state.

There are studies that indirectly addressed suicidal behavior using rsfMRI techniques [e.g., they mainly investigated relevant predictors of suicidal behavior such as hopelessness (13, 14)], and provided useful information about suicidality. Hopelessness is a powerful and informative psychological construct about suicidal behavior and addressed three major constructs: feelings regarding the future, loss of motivation, and expectations (15).

Interestingly, Northoff and colleagues (16) suggested that hopelessness and self-related processing were associated with higher resting-state neural activity in the DMN. Grimm and colleagues (17) aimed to investigate whether self-related emotional judgment may induce reduced negative BOLD responses (NBRs) in DMN regions of depressed patients. Reduced NBRs in the anterior regions of the DMN associated with abnormally increased self-focus together with ruminations, self-blame, abnormal coupling of the self to negative emotions, and enhanced attention to the own self were found among depressed patients relative to healthy individuals (18). Interestingly, higher self-relatedness of negative stimuli was also associated with hopelessness measured using the Beck Hopelessness Scale. This is in line with the observation that, clinically depressed patients usually present alterations in anticipating the future, but they also show increased retrieving and ruminating about past events (19). The study of Grimm and colleagues (17) postulated the existence of a dysregulated balance between anterior and posterior medial regions of NBRs during self-related judgment that may suggest a shift in the functional balance between anticipation and retrieval, or future and past.

**Figure 1:**
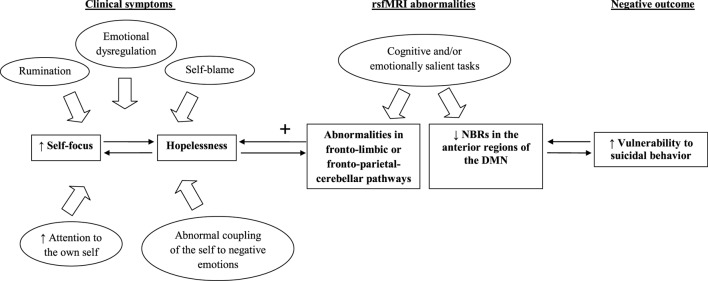
**The relationship between fMRI abnormalities and vulnerability to suicidal behavior**. Note: default mode network (DMN), negative blood oxygenation level-dependent responses (NBRs), resting-state functional magnetic resonance imaging (rsfMRI).

Figure 1 showed the potential of rsfMRI techniques to inform about vulnerability to suicidal behavior. We mainly focused on self-relatedness of negative stimuli that, according to Grimm and colleagues (17), are predominantly related to reduced NBRs in the anterior regions of the DMN and increased vulnerability to suicidal behavior, whereas we omitted to mention the role of motivational and consummatory anhedonia that are presumably associated with reward network dysfunctions [for more details about this topic, see the recent review of Lener and Iosifescu (20)].

Further additional details need to be discussed in this regard. Recently, Johnston and colleagues (21) also reported an abnormally increased hippocampal activity during loss events, which has been associated with self-reported depression and hopelessness in a sample of 20 patients with treatment-resistant MDD and 21 healthy controls. According to the assumptions of Deakin (22), the authors hypothesized that the failure to deactivate the hippocampus during loss events is mediated by the abnormal median raphe nucleus functioning, which is normally implicated in mediating resilience and is usually able to inhibit rehearsal of aversive memories. This is also confirmed by the “dorsal raphe nucleus-periaqueductal gray-amygdala-striatum” hypothesis about depressive illness, which has been initially postulated by Deakin and Graeff (23). This theory suggested that major depression is a complex condition including anxiety symptoms associated with the overactivity of dorsal raphe nucleus and its projections to the amygdala, helplessness related to the overactivity of periaqueductal gray, anhedonia associated with the overactivity of caudate/striatum as well as ruminations related to the underactivity of median raphe nucleus and its projections to the hippocampus (23).

Finally, there are also imaging evidence that investigated suicidality as a secondary emergence of disturbed experience of self in subjects with personality disorders. For example, borderline personality disorders (BPD) may be significantly associated with suicidal behavior as subjects with this psychiatric condition frequently exhibit recurrent suicidal threats, gestures, behavior, or self-mutilation (9, 24). In particular, Oumaya and colleagues (24) reported that self-mutilating suicide attempters may be more likely to experience feelings of depression and hopelessness, impulsivity, and affective instability, but they may also underestimate the lethality of suicidal behavior. Xu and colleagues (25), in the effort to identify an objective test to assist the clinical diagnosis of BPD, analyzed a sample of 21 patients with BPD and 10 healthy controls and reported that the most discriminating deficits between the two groups were located in the left medial orbitofrontal cortex, the left thalamus, and the right rostral anterior cingulate cortex. Other researchers (26) used rsfMRI to investigate changes in functional connectivity in a sample of 32 subjects with antisocial personality disorder (APD) and 35 healthy controls. Both functional and structural deficits of the precuneus, the superior parietal gyrus, and the cerebellum that may underlie the low arousal, high impulsivity, lack of conscience, cold-bloodedness, and decision-making deficits have been reported in subjects with APD when compared to healthy controls exposing them to a greater risk for suicide.

## Main Shortcomings and Future Perspectives

Overall, rsfMRI studies suggest the potential to identify functional intermediate phenotypes that may provide interesting information about suicide risk identification.

However, rsfMRI studies need to be considered in the light of several limitations. First, based on the current knowledge about this topic, it is unclear whether the reported abnormalities represent risk markers for suicide or are directly related to the course of illness as a result of disease processes. Unfortunately, a comprehensive understanding of the altered mechanisms involved at the level of dysfunctional brain networks in subjects at risk for suicide is still lacking.

In addition, rsfMRI studies are performed using inferences at the group level according to traditional statistical hypotheses and the predictive potential of the patterns may be unlikely generalized when applying to new individuals (25). Moreover, existing rsfMRI studies usually include relatively small and clinically heterogeneous samples (e.g., predominantly suicidal young adults) that may have seriously limited their statistical power. Furthermore, subject motion during the scan may significantly influence rsfMRI findings, although several methods have been proposed including approaches to reduce the impact of motion artifacts. The potential effect of psychoactive medications in rsfMRI studies cannot be ruled out as well. Almost all of the patients who have been recruited by the existing studies were on psychotropic medications due to ethical considerations. Studies may also lack detailed information regarding medication doses or the duration of treatment.

In conclusion, elucidating the functional deficits associated with specific network disturbances may help to clarify the pathophysiological mechanisms underlying suicidal behavior and assist in identifying high-risk individuals in clinical practice. However, further studies involving larger samples of non-medicated individuals are needed to investigate the nature of the relationship between rsfMRI data and suicidal behavior. These additional researches could focus on examining at-risk individuals and relatives of affected subjects or shifts in brain networks before and after the emergence of suicidal ideation/attempts in order to clarify specific causal pathways related to the observed network abnormalities. The utilization of multivariate algorithms to obtain information from multiple domains, such as neural networks, genetic and epigenetic markers, self-report instruments, clinical interviews, and support diagnosis and treatment selection, are recommended as well.

## Author Contributions

GS conceived, designed, and drafted the present manuscript. MPa participated in the concept and helps in reviewing the current literature about the main topic. MPo critically reviewed the paper. MA and PG provided the intellectual impetuous and supervised the writing of the manuscript. All authors approved the final version of the manuscript.

## Conflict of Interest Statement

The authors declare that the research was conducted in the absence of any commercial or financial relationships that could be construed as a potential conflict of interest. The reviewer JW and handling Editor declared their shared affiliation, and the handling Editor states that the process nevertheless met the standards of a fair and objective review.
